# Vitamin D and Periodontal Health: A Systematic Review

**DOI:** 10.7759/cureus.47773

**Published:** 2023-10-26

**Authors:** Monali Shah, Megha Poojari, Prasad Nadig, Dinta Kakkad, Sudeshna Banerjee Dutta, Susmita Sinha, Kona Chowdhury, Namrata Dagli, Mainul Haque, Santosh Kumar

**Affiliations:** 1 Periodontology, KM Shah Dental College and Hospital, Sumandeep Vidyapeeth, Vadodara, IND; 2 Public Health Dentistry, Gujarat University, Ahmedabad, IND; 3 Medical Surgical Nursing, Shri Anand Institute of Nursing, Rajkot, IND; 4 Physiology, Khulna City Medical College and Hospital, Khulna, BGD; 5 Pediatrics, Gonoshasthaya Samaj Vittik Medical College, Dhaka, BGD; 6 Dental Research, Karnavati School of Dentistry, Karnavati University, Gandhinagar, IND; 7 Research, Karnavati School of Dentistry, Karnavati University, Gandhinagar, IND; 8 Pharmacology and Therapeutics, National Defence University of Malaysia, Kuala Lumpur, MYS; 9 Periodontology and Implantology, Karnavati School of Dentistry, Karnavati University, Gandhinagar, IND

**Keywords:** cholecalciferol, nutrition, vitamins, gum disease, periodontal disease, periodontal health, well-being, mouth cavity, oral physiology, vitamin d

## Abstract

The role of vitamin D in maintaining gum well-being is crucial. However, scientific research reported that the connotations of cholecalciferol and periodontal health have been divested in the present literature. However, there is enormous heterogeneity in the data available. The current review aims to systematically review and appraise the available literature investigating the role of vitamin D in maintaining periodontal health. Studies included randomized controlled trials and clinical trials following Preferred Reporting Items for Systematic Reviews and Meta-Analyses (PRISMA) guidelines and cohort studies reporting associations between vitamin D and oral health in systemically healthy patients. Databases such as PubMed, Google Scholar, Scopus, Embase, and other sources, including hand search, were searched until May 2023 using together-equipped search sequences. Altogether, scientific articles that conform to the inclusion principles underwent a thorough eminence evaluation. All papers meeting inclusion criteria were subject to quality assessment, and the method used to assess the risk of bias was the Cochrane risk of bias tool. The search identified 1883 papers, among which 1435 were excluded after title evaluation. After abstract and title screening, 455 were excluded, and six full texts were assessed. After full-text evaluation, two articles were excluded, and only four were included. The data shows vitamin D's association with oral health maintenance. Along with its action on bone metabolism, it has extended function, which provides for its action as an anti-inflammatory agent and production of anti-microbial peptides, which help maintain oral health. Although the literature available is immense, there is enormous heterogenicity in the papers conducted to appraise the association between vitamin D and oral health. This systematic review has filtered all the data to review a few essential aspects of the role of vitamin D in maintaining oral physiology. Vitamin D has a linear relationship with periodontal health; however, the evidence is insufficient, and further studies must be done.

## Introduction and background

Periodontal health is a crucial indicator of overall health, the well-being of an individual, and the quality of life [1-3]. World Health Organization (WHO) described oral health as the ambiance of the buccal cavity, teeth, and orofacial edifices that empower people to execute fundamental activities like ingesting and swallowing food, maintaining respiration, and verbal communication [4]. It additionally involves mental factors like self-reliance, assertiveness, well-being, and the capacity to fraternize and work without enduring pain, discomfort, or embarrassment [5-9]. It has shown a strong association with many systemic [10] and non-communicable diseases (NCDs) [11] like hypertension [12, 13] and diabetes [14,15]. NCD incidence is increasing [16-19] worldwide due to lifestyle changes [20,21], environmental factors [22,23], and genetics [24-26]. Periodontal or buccal cavity disease is also called “the mirror” of the entire body, as many systemic diseases have oral manifestations [27].

Periodontium reinforces buccal anatomical structures, including tissues, essential to maintaining oral health [28]. Periodontal health is the nonexistence of histopathological confirmation of inflammation in the prop-up anatomy of the teeth and acts as the principal constituent for good oral health [29]. Any disease or pathology in the periodontium will, in turn, compromise oral health [29, 30]. It becomes crucial for an individual to maintain their periodontal health to lead a healthy life [31-33]. Well-balanced nutrition, nutritional supplements, and lifestyle alterations are strongly advocated to maintain good periodontal health [34]. These features are considered moldable factors to improve gum health. However, multiple correlations promote periodontal diseases, such as oral cleanliness, heritable and epigenetic aspects, and systemic health [34]. 

Vitamins are organic micronutrient particles indispensable for any living creature, including plants and animals, in minute amounts to confirm apposite metabolism, gene directive, and immune purposes [35]. They act as a catalyst for all metabolic reactions and are an integral element required for cell growth and maintenance [36-38]. Its disproportion led the way to undernourishment, affecting an individual's overall health [39]. Vitamin D is responsible for bone health and promotes Ca^2+^ absorption [40,41]. Ground-level of vitamin D in the blood diminishes Ca^2+^ absorption [42]. Thus, it stimulates to liberate extra amount of parathyroid hormone, osteoclastogenesis, and weakening Ca^2+ ^adhesion with bone to prevent serum hypocalcemia [42]. Vitamin D (also denoted as "calciferol") is a fat-soluble vitamin that is inherently available in a few foods, fortified to others, and obtainable as a dietetic supplement. The recommended dietary allowance (RDA) for a grown person aged 19 years and above is 600 IU (15 mcg) every day for males and females, and for adults > 70 years it is 800 IU (20 mcg) day-to-day [43]. The best source of vitamin D is cod liver oil, salmon, swordfish, tuna, orange juice fortified with vitamin D, dairy and plant milk fortified with vitamin D, sardines, beef liver, egg yolk, and fortified cereals. [43].

Vitamin D gets synthesized biologically when sunlight's ultraviolet (UV) rays directly penetrate the surface of human skin and start manufacturing vitamin D [44]. There are not plenty of foods that intrinsically comprise vitamin D [45,46]. Still, fatty fish like salmon, mackerel, and herring and fish oils like cod liver oil do [47]. Common vegan sources of vitamin D are mushrooms, invigorated plant-based kinds of milk, exhilarated orange juice, cheese, refreshed tofu, reinforced breakfast cereals, and yogurt [48]. Vitamin D is generically divided into vitamin D2 and D3 [49]. Vitamin D2 is synthesized by the grace of ultraviolet radiation of ergosterol from yeast [50,51], and vitamin D3 results from ultraviolet exposure of 7- dehydrocholesterol from lanolin, revealing the biological endeavor of cholecalciferol (vitamin D3), which is produced in the human sun-exposed cutaneous area [52,53]. The extensively recognized biomarker study for vitamin D level is the estimation of serum 25-hydroxyvitamin D (25[OH]D) [54-56]. This conversion procedure of inactive vitamin D to active happens in 2 phases: (a) inside the liver, cholecalciferol is hydroxylated to 25-hydroxycholecalciferol (25[OH]2D) by the enzyme 25-hydroxylase, and (b) in the kidneys, 25-hydroxycholecalciferol is transformed to 1,25(OH)2D by the enzyme 1α hydroxylase [57,58].

Vitamin D performs similarly to endocrine messenger molecules primordially and regulates intestinal absorption to support serum Ca^2+^ and phosphate equilibrium [59]. Furthermore, vitamin D controls cell distinction, evolution, and intrinsic immune arrangement; it performs as an autocrine and paracrine biomolecule [60-64]. Further, the Vitamin D receptor (VDR) is a receptor particle that is a nuclear receptor (NR) superfamily colleague. It holds together to activate Vitamin D and umpires’ biological actions through ﻿ induction or repression of gene transcription [57,65]. Also, VDRs can fasten (together) a considerable crowd of the genomic site and modulate the utterance of diverse principal earmark genes [66]. Vitamin D is an elemental membrane-associated protein for its nongenomic products (communication passageways) [67-69]. Multiple studies revealed that vitamin D is responsible for a broad range of physiological functions [50,70,71] because it regulates the expression of many genes and their biotic course [72,73]. It has been reported that Vitamin D exerts a straightforward influence on the epigenome and the manifestation of over 1000 genes in utmost hominoid tissues and cell categories [72]. Vitamin D affects bone, antimicrobial action, and anti-inflammatory effects, maintaining oral health [55,74,75]. Vitamin D insufficiency is the most common global medical disorder. Around one billion population around the globe are suffering from lack or inadequate vitamin D. The prevalence of vitamin D deficiency among grown-up people was stated to be 14-59% with a greater pervasiveness in Asian nations [76-78]. Once a patient is deficient in Vitamin D, it is recommended to take a minimum of 8 weeks to correct the optimum level of serum cholecalciferol, either 6,000 IU daily or 50,000 IU weekly [79-82].

Worldwide awareness-building program regarding the importance of vitamin D has augmented aggressively due to the prevalence of its deficiency [78,83-86]. Major periodontal diseases are multiplex and involve diverse causes [87,88]. The function of vitamin D in conserving overall mouth cavity well-being [55,75]. Vitamin D preserves bone health and metabolic activities that promote the health of soft tissues [30,89,90]. The medical perspective regarding the role of vitamins in maintaining and promoting health is well established [91-93]; however, its correlation with oral health has very heterogeneous literature. Available papers focus more on the correlation with disease or pathology and adjustment of vitamin D dosage [94-96] rather than defining vitamin D's association with periodontal health. Multiple studies reported that a patient without clinical attachment loss (CAL) of tooth or mandibular and maxillary bone damage and with minimized periodontium in either a non-periodontitis or recovered periodontitis case is considered clinical good gingival health with intact and stable periodontium [96-100].

Problem statements

The research question is to appraise the outcome of vitamin D addition to the improvement of periodontal parameters in humans with unhealthy periodontium and to evaluate the association between serum vitamin D levels and periodontal health factors. It has been reported that any degrees of raise in standard deviation (SD) of the log-transformed intensity of cholecalciferol emanated in a 15% fall-out in the threat of periodontitis "[OR=0.85, 95% confidence interval (CI): 0.78-0.93, p=0.006]" subsequent to multivariable adaptation [95].

﻿Objectives of this study

The objectives of this systematic review are to evaluate the impact of vitamin D on periodontal health by synthesizing the available evidence from relevant studies and examine effect of different dosage of vitamin D on periodontal health outcomes, to inform and provide evidence-based review to healthcare providers and policymakers about the potential effect of vitamin D on periodontal health and the implications for public health strategies, to identify gaps in the existing literature and highlight areas where additional exploration is required to understand the vitamin D-periodontal health connection better.

## Review

Material and methods

This systematic review follows the Preferred Reporting Items for Systematic Reviews and Meta-Analyses (PRISMA) guideline.

Eligibility Criteria

Inclusion criteria: This review encompassed randomized controlled trials (RCT) and clinical trials reporting vitamin D supplementation and its effect on periodontal health. Only human studies published until May 2023 were included. Electronically published as well as printed journal articles were incorporated. Prospective cohort studies evaluating periodontal parameters as their primary or secondary outcomes were included.

Exclusion criteria: Interventions done in the study that are not measurable for vitamin D. Studies having less than 8 weeks of follow-up.

Information Sources

Electronic databases like PubMed, Google Scholar, Embase, and Scopus were scoured to shortlist the articles.

Search Plan

The search approach included Medical Subject Headings (MeSH) terms and keywords: “Vitamin D and Periodontal Health” and “Vitamin D and Periodontal Diseases” due to the heterogenicity of articles and to limit the search to the specified topic. Cross-referencing was done to exploit the references of full-text articles. Published papers were also searched using hand search.

Study Selection Process

All the repetitive or matching papers were eliminated after associating the results from the different research strategies. Two authors (MRS and MEP) individualistically scrutinized all the articles' abstracts obtained after the search strategy. After that, all the scientific literature that met the encompassing indicator was searched for full text. The researchers self-reliantly evaluated all the shortlisted articles after abstract evaluation to ascertain whether this article should or should not be integrated into this systematic review.

Data Gathering Procedure

Two authors (MRS and MEP) collected data independently; then, data or necessary information was taken out according to improvised planning. Masking of journal titles and authors' names was not carried out. Data extraction was done in tables, where each article was mentioned along with its essential aspects, including the type and country of research, age, number of participants, relationship between vitamin D and periodontal diseases, follow-up period, and statistical significance. After data searching and recording for each paper was completed, the following steps were conducted: (a) publication year and title in short, (b) country and type of research carried out, (c) details of participants at baseline, (d) interventions applied, (e) parameters assessed and the timeline for the study, (f) bias related data, and (g) results in detail dividing each parameter.

Assessment of Bias Throughout the Research Papers

The risk of bias (RoB) evaluation was shepherded by two authors (MRS and MEP). The procedural excellence of the integrated study was counted, conferring to the quality appraisal instrument developed by Cochrane's RoB tool. The tool included domains to evaluate selection bias due to random sequence generation, selection bias due to apportionment camouflage, recording bias, implementation bias, finding bias, attenuation bias, and additional bases of preference or bias; for each domain, the assessment was given as high, low, or unclear as per the guidelines. Disagreements between authors were resolved by consensus with other authors.

Results

Study Selection

**Figure 1:**
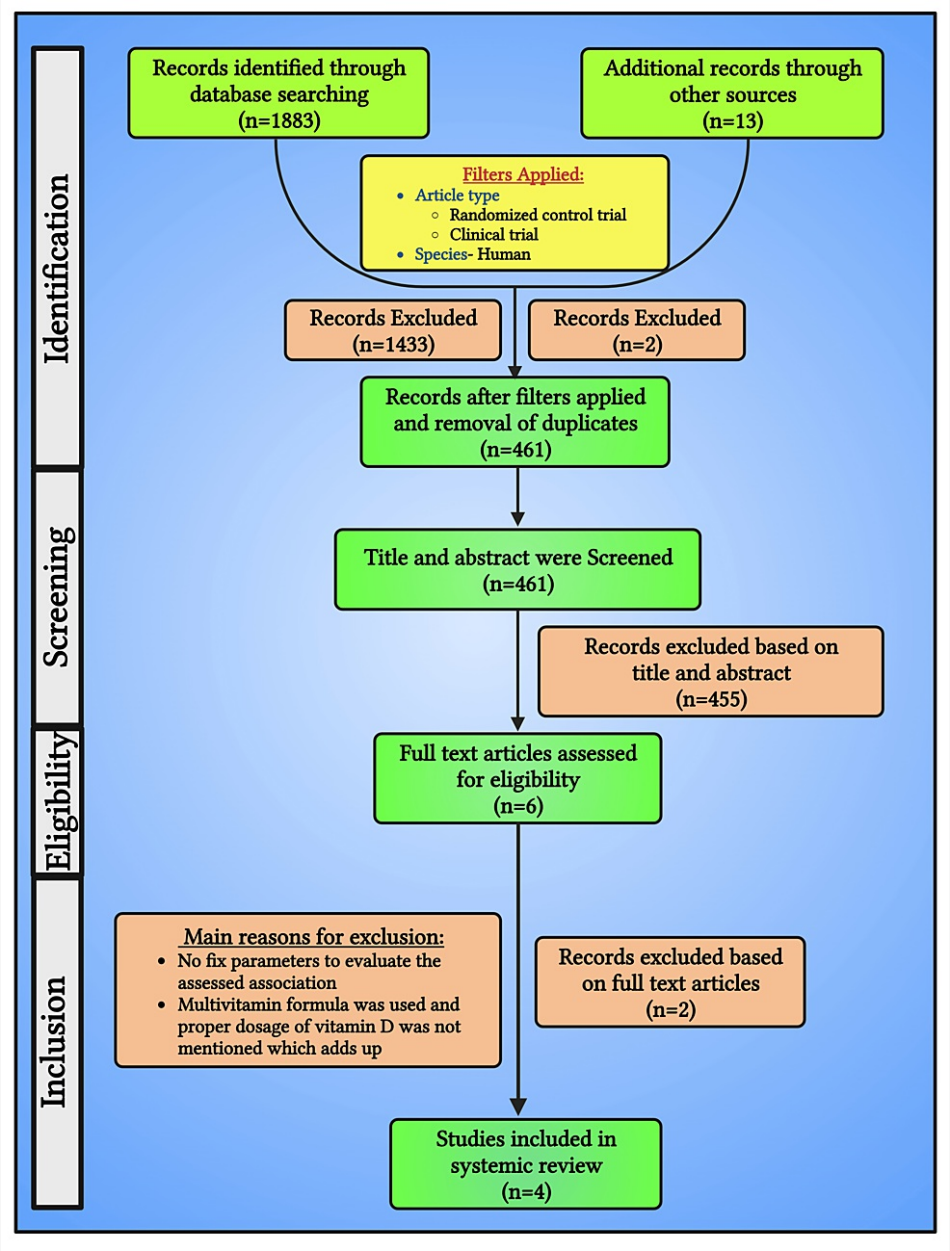
A simplified PRISMA flow chart showing the literature search PRISMA: Preferred Reporting Items for Systematic Reviews and Meta-Analyses This figure has been drawn with the premium version of BioRender (https://biorender.com/ accessed on October 11, 2023) with the license number XP25YIQG2X. Image credit: Susmita Sinha

Search engines PubMed, Google Scholar, Embase, and Scopus, identified 1883 papers after applying MESH terminologies. A total of 1433 articles were excluded after applying filters. Filters used were (a) article type: randomized controlled trials and (b) species: humans. Four hundred and fifty articles underwent title evaluation by both authors independently. About 13 articles were included using a manual search. Two articles were excluded as duplicates, and 11 were subjected to further assessment. The remaining 461 articles, consisting of electronic and manual searches, were screened using titles and abstracts. After title and abstract screening, 455 records were excluded. A total of 6 full-text articles were evaluated for eligibility, among which 4 were encompassed in this systematic review (Figure 1).

Two more articles congregated the enclosure principles; however, they were excluded after full-text evaluation, as the study conducted by Dixon et al. [99] conducted a convenience survey that included a questionnaire, and no fixed parameters were assessed. Another RCT was done by Harpenau et al. [100] in which a multi-vitamin formula was used, and the proper dosage of vitamin D was not mentioned, which adds confounding factors to the results to rule out any association between periodontal health and vitamin D (Table 1).

**Table 1:** Excluded articles

Serial No.	Author and Year	Title in Short	Type of Research	Reason for Exclusion
1	Dixon et al. (2009) [99]	Usage of Ca^2+^ and vitamin D in periodontal maintenance	Convenience survey	As it includes a questionnaire for evaluation and no fixed parameters are assessed
2	Harpenau et al. (2011) [100]	Effect of nutritional supplement on periodontal parameters	Randomized controlled trial	Multi-vitamin formula is being used, and proper dosage of vitamin D is not mentioned in order to rule out any association with vitamin D

Study Characteristics

Three RCTs and one prospective cohort study were included. The studies were done in 2011, 2016, and 2020. Two studies were done in Germany, one in India, and one in the USA. The age range of these studies was 18-75 years. The number of participants included was about 18-28 in three studies; however, one study had 96 participants. Hiremath et al. [101] used different vitamin D dosages, including 2000 IU, 1000 IU, and 500 IU, as an intervention. The Woelber et al. [102] study had a healthy diet as an intervention like Tennert et al. [103], where a diet low in carbohydrates and rich in omega-3 fatty acids, vitamins C and D, antioxidants, and fiber was used. In the prospective cohort study, the intervention included vitamin D supplements and periodontal maintenance every three months [104]. Outcomes assessed included clinical parameters like gingival scores, bleeding index, plaque index, calculus index, probing depth, clinical attachment loss (CAL), bleeding upon probing (BOP), periodontal Inflamed surface area (PISA), furcation involvement; microbiological parameters like total bacterial count and specific bacterial count; radiographic parameters like alveolar crest height (ACH) and nutrient analysis. In the follow-up period, they were ranged from 8 to 48 weeks (Table 2). 

**Table 2:** Study characteristics of included studies

Serial No.	Author and Year	Country	Type of Research	Age (Number Of Participants)	Interventions	Outcome	Follow-up
1	Tennert et al.(2020)[103]	Germany	Randomized control trial (RCT)	18 to 75 (16)	Healthy Diet; Standard Diet	Total bacterial counts, aerobic and anaerobic bacterial count, specific species count: Capnocytophaga spp., Granulicatella adiacens, Fusobacterium spp., Actinomyces spp., Streptococcus mitis cluster	8 weeks
2	Hiremath et al. (2011) [101]	India	RCT	18 to 64 (96)	Group A-Vit D 2000 IU; Group B-Vit D 1000IU; Group C-Vit D 500IU; Group D-Placebo	Serum Vitamin D level, Serum Ca^2+^ level, Gingival scores, Bleeding Index	8 weeks 4 days
3	Woelber et al. (2016) [102]	Germany	RCT	Experiment Group: 23 to 70 years (10) Control Group: 24 to 63 years (5)	Experiment Group: Food régime rich in omega-3 fatty acids, ascorbic acid, fat-soluble seco-sterols such as calciferol, which inhibits reactive oxygen species, and fiber. However, a tiny portion of carbohydrates consumed; Control Group: no modification in diet	Plaque index, gingival index, probing depths, losing clinical bonding of tooth, and hemorrhage during examination probe, especially inflamed periodontal surface area	8 weeks
4	Garcia et al. (2011) [104]	USA	Prospective Cohort	Mean Age (No. of patients): Vitamin D Takers: 64 (23); Vitamin D Non-Takers: 62 (28). Takers: Regular consumption of Ca^2+^(‡1,000 mg/day) and vitamin D (‡400 IU/day) as supplements, with their regular healthy diets, for above 18 months at the time of their initial visits and findings. Non-Takers: Not been taking either vitamin D or Ca^2+^ supplementation and had dietary intakes of vitamin D and Ca^2+^ 400 IU/day and below 1,000 mg/day, respectively.	Periodontal maintenance at 3-month interval	Nutrient analysis (NHANES-II and NHANES-III) gingival index, plaque index, probing depth, attachment loss (AL), bleeding on probing, calculus index, and furcation involvement, Radiographic assessment (Alveolar crest height)	24 weeks and 48 weeks

Risk of Bias in Research

**Figure 2:**
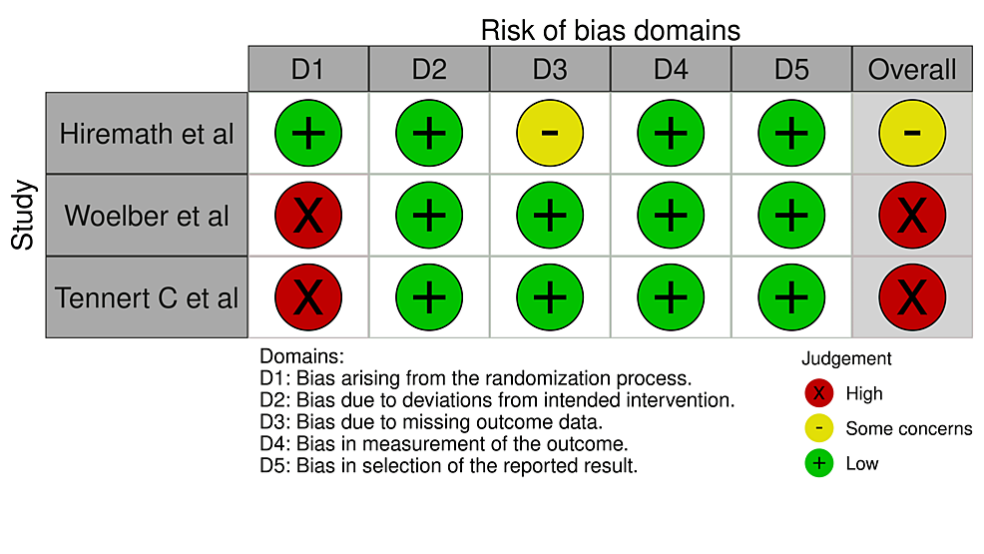
Traffic light plot for risk of bias of included RCTs RCT: Randomized control trial Hiremath et al. 2011 [101], Woelber et al. 2016 [102], Tennert et al. 2020 [103]

**Figure 3:**
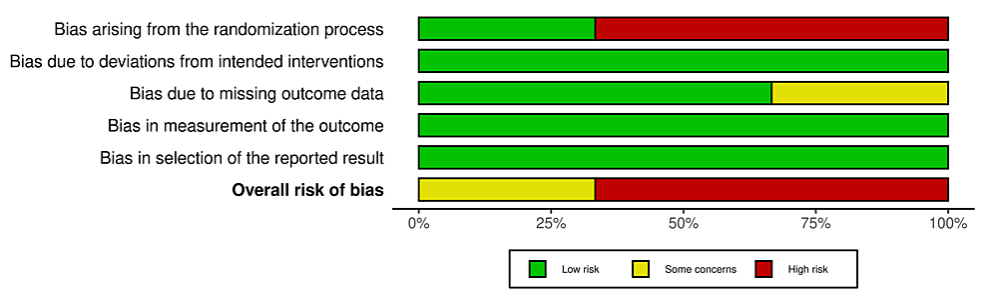
Summary plot for risk of bias of included RCTs RCT: Randomized control trial Hiremath et al. 2011 [101], Woelber et al. 2016 [102], Tennert et al. 2020 [103]

Two RCTs, Tennert et al. 2020 [103] and Woelber et al. 2016 [102], had a high RoB; however, the research conducted by Hiremath et al., 2011 [101] showed some concerns. The details of the RoB are depicted in Figure 2 and Figure 3.

Results of Individual Studies

In the first study [103], at 8 weeks of follow-up, 16 participants were assessed. The results showed statistically insignificant results in inter and intragroup comparison in terms of total bacterial count in saliva, specific bacterial count in plaque, and aerobic and anaerobic bacterial count. Regarding species count in plaque, a statistically significant difference was seen in the healthy faction plaque sample of *Fusobacterium spp*. (p=0.03500), S*treptococcus mitis* group (p=0.025), *Actinomyces spp*. (p=0.02), and *Granulicatella adiacens *(p=0.019). Considering the specific species count in saliva, a statistically significant decrease in intragroup comparison of the diet containing low-calorie and high-roughage consumers was observed (Table 3).

**Table 3:** Results of included studies BOP: Bleeding upon probing; PISA: Periodontal inflamed surface area

Serial No.	Author and Year	Number of Participants/ Follow-up	Results			
			Total bacterial count in plaque (median)	Aerobic and anaerobic bacterial count (median)		
1	Tennert et al. (2020) [103]	N=16. 8 weeks		At baseline	Fusobacterium spp. (p=0.035), Streptococcus mitis crowd (p=0.025), Granulicatella adiacens (p=0.019), and Actinomyces spp. (p=0.02)	
				Control group: 10.21/9.55 log10 CFU/ml		
			Healthy diet cluster: 3.4 × 1010 CFU/ml	Healthy diet assembly: 10.03/9.84 log10 CFU/ml		
			At 8 weeks	At 8 weeks		
			The control clutch was 1.8 × 1011 CFU/ ml	Control group: 9.23/8.89 log10 CFU/ml; P>0.05		
			Healthy diet faction 1.4 × 1010 CFU/ml; P > 0.05	Healthy diet group: 9.31/9.18 log10		
							Serum Vitamin D level	Gingival scores		
2	Hiremath et al. (2011) [101]	N=96. 8 weeks 4 days	Per month analysis	Mean, At baseline		1
			Group A: 9.9116 ng/ml	Group A: 2.41±0.54		
				Group B: 2.39±0.57		
				Group C: 2.24±0.46		
				Group D: 2.23±0.61		
			Group B: 5.6248 ng/ml	At 90 day		
			Group C: 4.2743 ng/ml	Group A: 0.34±0.60		
				Group B: 0.55±0.66		
				Group C: 0.88±0.98		
				Group D: 1.89±0.64		
			Group D: 0.1156 ng/ml	Group A, B, and C: p<0.0001 and Group D: p>0.05		
			Group A, B, and C: p<0.001 and Group D: p>0.05			
			Plaque Index (PI) and Gingival Index (GI) (Mean±SD)	BOP and PISA	Plaque Index and Gingival Index (Mean ± S.D)	
3	Woelber et al. (2016) [102]	N=15. 8 weeks	Experiment (E) Vs. Control (C)	Experiment/Control		
			Week 1: PI: E: 0.77±0.52/C: 0.75±0.63 and GI: E: 1.10±0.51/C:1.01±0.14	Week 2: 53.57±18.65/46.46±15.61 and 638.88±305.41/666.24±420.05		
			Week 8: PI: E - 0.84±0.47/C - 0.97±0.70 and GI: E - 0.54±0.30/C - 1.22±0.17	Week 8: 24.17±11.57/64.06±11.27 and 284.83±174.14/963.24±373.78		
			Plaque Index: p=0.084 (Experimental Vs. Control Group between Week 1 and 8)	BOP: p=0.012		
			Gingival Index: p<0.001 (Experimental Vs. Control Group between Week 1 and 8)	PISA: p<0.001		
			Nutrient analysis	Gingival Index and BOP	Plaque Index and Calculus Index	Probing depth and Attachment loss
4	Garcia et al. (2011) [104]	N=51. 24 and 48 weeks	Mean in Takers	In Takers/Non-Takers	In Takers/Non-Takers	In Takers/Non-Takers
			Daily Ca^2+^ Intake: 1,769 mg; Daily vitamin D intake: 1,049 IU	At baseline: 0.7/1.0 and 0.70/0.75.	At baseline: 0.80/0.96 and 0.20/0.25	At baseline: 2.2/2.35 AND 1.8/2.0
			Mean in Non-takers	At 12 months: 0.4/0.6 and 0.54/ 0.56	At 12 months: 0.75/0.65 and 0.01/0.01	At 12 months: 1.7/1.9 and 1.25/1.45
			Daily Ca^2+^ Consumers: 642 mg; Daily Vitamin D Intake: 156 IU	Gingival Index: p<0.0001/p=0.002	Plaque Index: p<0.0001/p=0.002	Probing Depth: p<0.0001/p=0.002
			p<0.0001	BOP: p<0.0001/p=0.002	Calculus Index: p<0.0001/p=0.002	Attachment Loss: p<0.0001/p=0.002

In the second study [101], at 60 days of follow-up, 96 participants were assessed. The outcomes revealed a statistically significant escalation in groups taking vitamin D add-ons (p<0.001) and insignificant slender rises among those having inactive medication (sugar pill) clutch (p>0.05) in terms of serum calciferol level. Considering the gingival scores, a statistically highly significant reduction in groups taking cholecalciferol supplementation (p<0.0001) on subsequent visits depending on the dosage, while statistically insignificant results were seen in the placebo-consuming folk (Table 3).

In the third study [102], at 8 weeks follow-up, 15 participants were assessed. The results showed statistically insignificant results regarding plaque index, probing depth, and CAL. The statistically significant variance was observed in relationships of gingival index (p<0.001) and BOP (p=0.012). The statistically highly significant difference was seen in terms of periodontal inflamed surface area (PISA) (p<0.001) (Table 3).

In the fourth study [104], at 48 weeks follow-up, 51 participants were assessed. Overall, the non-takers had worse clinical outcomes than the takers at baseline, and this pattern mostly continued throughout the research. Periodontal measurements were, on average, 23%, 19.9%, and 15.6% better in takers at initial value before intervention, 6 months, and 12 months compared to non-takers. Probing depths in healthy gingiva typical spectrum are 1-3 mm. A penetration of more than 3 mm is imaginable for distress apprehension [105]. Conventionally, clinical periodontal appraisal techniques involve pocket probing depth (PPD), bleeding on probing (BOP), clinical attachment level (CAL), and radiological evaluation of the alveolar bone volume, which are extensively utilized and recognized [106,107]. None of the clinical and roentgenographic test results were statistically substantial in univariate analysis due to relatively large standard deviations. While there were no significant disparities between the groups in the radiographic measurements of variations in ACH at 6 and 12 months, X-ray optical density clients had denser bone than non-takers (P=0.07) (Table 3). 

**Figure 4:**
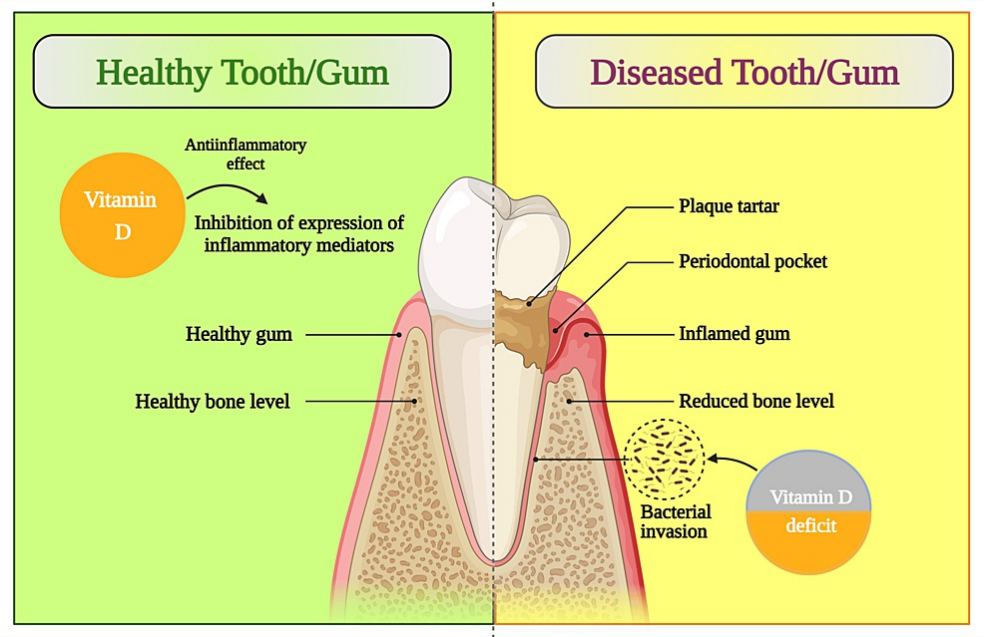
Schematic diagram showing the protective effects of vitamin D on periodontal health This figure has been drawn with the premium version of BioRender (https://biorender.com/ accessed on October 9, 2023) with the license number NW25YA0QJR. Image Credit: Susmita Sinha

An appropriate, well-adjusted food régime is indispensable to promote a healthy and quality life [108]. Each component of a balanced diet has certain specified functions, and it balances the overall health. Vitamins cover a small portion of the balanced diet chart but play an essential role [109-112]. Vitamin D promotes bone health and has certain extended functions [113-115]. Vitamin D enhances the host immune system [116] by reducing inflammation [117] and pathogenic microbial load, especially in the buccal cavity﻿, by synthesizing cathelicidin and β-defensin through activating keratinocytes, monocytes, and macrophages of the periodontal tissues [118]. Vitamin D alters the complex metabolic pathways of pathogens, lowering the infective microbes' metabolic activity [119-122]. As discussed by Tennert et al. 2020 [103], a diet rich in vitamin D leads to a reduction of gingival and periodontal inflammation (Figure 4), and it also alters the composition of plaque by reducing the count of pathogenic bacteria, consecutively improving the periodontal health [123].

The anti-inflammatory action of vitamin D is well discussed in the literature. However, the relationship of dosage or amount of vitamin D required to maintain adequate periodontal health is still not determined. Periodontal diseases are usually multifactorial, and vitamin deficiency is a factor that could affect periodontal health. There is no consensus about using vitamin D as a health-preventive agent for oral health. However, research steered by Hiremath et al. [101] exhibited that the anti-inflammatory action of vitamin D can be seen in doses 500-2000 IU. They also demonstrated that vitamin D dosage was directly proportional to the period to achieve the anti-inflammatory effect, i.e., the higher the dosage, the earlier the anti-inflammatory effect. Vitamin D-deficient patients can benefit from oral supplements for 2-3 months [101]. Another study reported that the ideal 25(OH)D3 acclaimed strength in blood plasma for osseous mass is not below 80 nmol/L for periodontic anatomical structure around 90-100 nmol/L [124]. Persistent low (>90-100 nmol/L) amounts of vitamin D aggravate periodontal disease progression resulting in tooth loss [125,126].

**Figure 5:**
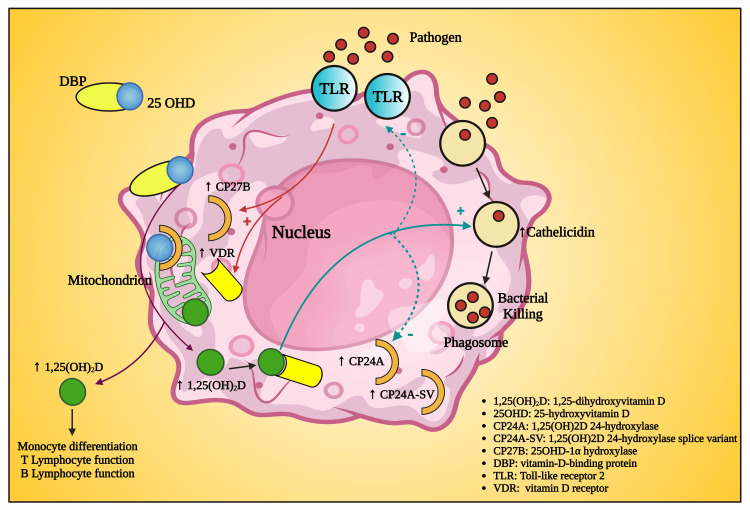
Schematic diagram showing the role of vitamin D in innate immunity This figure has been drawn with the premium version of BioRender (https://biorender.com/ accessed on October 9, 2023) with the license number BE25Y9UD7X. Image Credit: Susmita Sinha

The effect of vitamin D is usually thought to be limited to the development and maintenance of bone [90,127]; however, vitamin D deficiency is associated with acute (respiratory tract infections), chronic inflammatory and metabolic diseases like type I diabetes mellitus (T1DM), type 2 diabetes mellitus (T2DM), insulin resistance, rheumatoid arthritis (RA), obesity, inflammatory bowel disease (IBD), Alzheimer's disease, metabolic syndrome, cancer, osteoporosis, and cancer [128,129], which is related with periodontal diseases [114]. In the currently available literature, there is a cornucopia of evidence on the role of vitamin D as a potent regulator of innate immunity response (Figure 5) [130-133]. Vitamin D, when upregulated by toll-like receptors, the cells of innate immunity produce 1,25(OH)3D3 intracellularly, which releases cathelicidin (A 3^rd^ generation epithelial antimicrobial peptide) [71,134-136].

One of the most common signs of periodontal inflammation is bleeding on probing (BOP) [137]. Periodontal health is an absence of active periodontal inflammation, i.e., lack of bleeding or probing. Lang and Bartold defined periodontal health originally defined as the non-existence of histological substantiation of periodontal inflammatory condition and no affirmation of anatomical negative alteration to the periodontium [29]. Mariotti and Hefti defined that periodontal health was often an afterthought and was defined as the absence of the signs and symptoms of a periodontal disease. Accordingly, these strict and sometimes disparate definitions of periodontal disease have resulted in an idealistic requirement of a perfect periodontium for periodontal health, which makes us all diseased in one way or another [138].

A study by Garcia et al. 2011 [104] concluded that there was less BOP and less inflammation in patients undertaking vitamin D supplements. A linear association between inflammation and vitamin D can be drawn owing to the antimicrobial activity of vitamin D [138,139]. Vitamin D fortifications can minimize the harshness of periodontal disease and can be used as a modest selection for maintaining periodontal health [140]. There are numerous articles about the affiliation between vitamin D and periodontal health, so health maintenance recommendations for vitamin D intake have been made [121,141-143]. Still, most of the population does not meet the daily intake range [144]. Vitamin D deficiency is growing [78,145-148], and it becomes essential to include daily vitamin D supplementation to maintain health.

The number of studies available on vitamin D's association with periodontal health is enormous. However, the type of studies conducted has multiple interventions, which could act as confounding factors. Most studies have small sample sizes, affecting the study's power. The parameters that are assessed and the type of population included have huge heterogenicity, due to which meta-analysis was not carried out. Maintaining periodontal health to maintain an individual's overall health is paramount. All available data contemplated the potential benefits of vitamin D on periodontal health. Hence, a dietary protocol can be adopted by clinicians to implement on the patients in daily practice. A diet rich in vitamin D can positively affect patients' periodontal health when taken in optimum dosage.

Limitations

Although in determining the efficacy of interventions RCTs are essential research work. Nevertheless. a shortcoming to using RCTs in population health studies is the deficiency of generalizability or subdued outer validity. Additionally, it is frequently small and/or too short a duration for uncommon harms. Moreover, RCTs are expensive, laborious, time-consuming, and complicated to design, implement, and monitor [149,150]. We have selected RCTs for this systematic review. The systematic review has its inherent trouble, such as hazards of bias, such as selection bias, insufficient blinding, abrasion bias, and selective outcome reportage; a discrepancy that comprises clinical or statistical heterogeneousness; and inaccuracy that can lead to Type I and Type II errors [151].

## Conclusions

There is a linear association between vitamin D and periodontal health. Yet, the association needs to be confirmed with more longitudinal studies with larger sample sizes focusing on these two parameters. The antimicrobial function of vitamin D is of more importance than its function on bone health maintenance owing to the care of periodontal health. The detailed anti-microbial mechanism of vitamin D in maintaining health needs to be studied further as the data available is discrete and non-specific. Dietary supplements are usually limited and sparse; added vitamin D can potentially induce beneficial effects on periodontal health.

## References

[1] Vivek B, Ramesh KS, Gautami PS, Sruthima GN, Dwarakanath C, Anudeep M (2021). Effect of periodontal treatment on oral health-related quality of life - A randomised controlled trial. J Taibah Univ Med Sci.

[2] James A, Janakiram C, Meghana RV, Kumar VS, Sagarkar AR, Y YB (2023). Impact of oral conditions on oral health-related quality of life among Indians- a systematic review and Meta-analysis. Health Qual Life Outcomes.

[3] Block C, König HH, Hajek A (2022). Oral health and quality of life: findings from the Survey of Health, Ageing and Retirement in Europe. BMC Oral Health.

[4] (2023). Oral health. https://www.who.int/news-room/fact-sheets/detail/oral-health.

[5] Goyal S, Gupta G, Thomas B, Bhat KM, Bhat GS (2013). Stress and periodontal disease: the link and logic!!. Ind Psychiatry J.

[6] Chimbinha ÍGM, Ferreira BN, Miranda GP, Guedes RS (2023). Oral-health-related quality of life in adolescents: umbrella review. BMC Public Health.

[7] Kisely S (2016). No mental health without oral health. Can J Psychiatry.

[8] Kisely S, Sawyer E, Siskind D, Lalloo R (2016). The oral health of people with anxiety and depressive disorders - a systematic review and meta-analysis. J Affect Disord.

[9] (2023). Action plan for oral health in South-East Asia 2022-2030. https://iris.who.int/bitstream/handle/10665/363753/9789290210061-eng.pdf?sequence=1.

[10] Nazir MA (2017). Prevalence of periodontal disease, its association with systemic diseases and prevention. Int J Health Sci (Qassim).

[11] Wolf TG, Cagetti MG, Fisher JM, Seeberger GK, Campus G (2021). Non-communicable diseases and oral health: an overview. Front Oral Health.

[12] Li Y, Yuan X, Zheng Q (2023). The association of periodontal disease and oral health with hypertension, NHANES 2009-2018. BMC Public Health.

[13] Muñoz Aguilera E, Suvan J, Orlandi M, Miró Catalina Q, Nart J, D'Aiuto F (2021). Association between periodontitis and blood pressure highlighted in systemically healthy individuals: results from a nested case-control study. Hypertension.

[14] Păunică I, Giurgiu M, Dumitriu AS, Păunică S, Pantea Stoian AM, Martu MA, Serafinceanu C (2023). The bidirectional relationship between periodontal disease and diabetes mellitus-a review. Diagnostics (Basel).

[15] Costa R, Ríos-Carrasco B, Monteiro L, López-Jarana P, Carneiro F, Relvas M (2023). Association between type 1 diabetes mellitus and periodontal diseases. J Clin Med.

[16] Budreviciute A, Damiati S, Sabir DK (2020). Management and prevention strategies for non-communicable diseases (NCDs) and their risk factors. Front Public Health.

[17] Chobe M, Chobe S, Dayama S, Singh A, Metri K, Basa JR, Raghuram N (2022). Prevalence of non-communicable diseases and its associated factors among urban elderly of six Indian states. Cureus.

[18] Wu BW, Skidmore PM, Orta OR (2016). Genotype vs. phenotype and the rise of non-communicable diseases: the importance of lifestyle behaviors during childhood. Cureus.

[19] Swarnakar R, Yadav SL (2022). Communicable to non-communicable disease pandemic in the making: an urgent call for post-COVID-19 preparedness. Cureus.

[20] Li Y, Fan X, Wei L, Yang K, Jiao M (2023). The impact of high-risk lifestyle factors on all-cause mortality in the US non-communicable disease population. BMC Public Health.

[21] Kelishadi R (2019). Life-cycle approach for prevention of non communicable disease. Adv Exp Med Biol.

[22] Prüss-Ustün A, van Deventer E, Mudu P (2019). Environmental risks and non-communicable diseases. BMJ.

[23] Dhimal M, Neupane T, Lamichhane Dhimal M (2021). Understanding linkages between environmental risk factors and noncommunicable diseases-a review. FASEB Bioadv.

[24] Tabatabaiefar MA, Sajjadi RS, Narrei S (2019). Epigenetics and common non communicable disease. Adv Exp Med Biol.

[25] Jamaluddine Z, Sibai AM, Othman S, Yazbek S (2016). Mapping genetic research in non-communicable disease publications in selected Arab countries: first step towards a guided research agenda. Health Res Policy Syst.

[26] Bronson SC, Seshiah V (2021). Transgenerational transmission of non-communicable diseases: how to break the vicious cycle?. Cureus.

[27] Varoni EM, Rimondini L (2022). Oral microbiome, oral health and systemic health: a multidirectional link. Biomedicines.

[28] Torabi S, Soni A (2023). Histology, periodontium. StatPearls [Internet].

[29] Lang NP, Bartold PM (2018). Periodontal health. J Periodontol.

[30] Könönen E, Gursoy M, Gursoy UK (2019). Periodontitis: a multifaceted disease of tooth-supporting tissues. J Clin Med.

[31] Malik R, Thanveer K, Singh V, Jain A, Mitra S, Singh S (2023). Impact of dental treatment on oral health-related quality of life of patients. Cureus.

[32] Kumari M, Patthi B, Singla A, Abdul HN, Mansoor MA, Rajeev A (2023). Assessment of oral health-related quality of life using the oral impact on daily performance (OIDP) instrument among secondary school teachers of Modinagar, Uttar Pradesh: a cross-sectional study. Cureus.

[33] Nayan K, Khan AA, Kusum P, Kumar P, Kumari L, Srivastav SK (2022). Utilization of dental care, tooth loss, and oral health-related quality of life in older adults visiting dental care centers in Indian settings. Cureus.

[34] Najeeb S, Zafar MS, Khurshid Z, Zohaib S, Almas K (2016). The role of nutrition in periodontal health: an update. Nutrients.

[35] Upadhaya SD, Kim IH (2020). Importance of micronutrients in bone health of monogastric animals and techniques to improve the bioavailability of micronutrient supplements - A review. Asian-Australas J Anim Sci.

[36] Parra M, Stahl S, Hellmann H (2018). Vitamin B₆ and its role in cell metabolism and physiology. Cells.

[37] Ofoedu CE, Iwouno JO, Ofoedu EO (2021). Revisiting food-sourced vitamins for consumer diet and health needs: a perspective review, from vitamin classification, metabolic functions, absorption, utilization, to balancing nutritional requirements. PeerJ.

[38] Lyon P, Strippoli V, Fang B, Cimmino L (2020). B vitamins and one-carbon metabolism: implications in human health and disease. Nutrients.

[39] Kraemer K, Gilbert C (2013). Do vitamin A deficiency and undernutrition still matter?. Community Eye Health.

[40] Khazai N, Judd SE, Tangpricha V (2008). Calcium and vitamin D: skeletal and extraskeletal health. Curr Rheumatol Rep.

[41] Sunyecz JA (2008). The use of calcium and vitamin D in the management of osteoporosis. Ther Clin Risk Manag.

[42] Nascimento GG, Leite FR, Gonzalez-Chica DA, Peres KG, Peres MA (2022). Dietary vitamin D and calcium and periodontitis: a population-based study. Front Nutr.

[43] (2023). The nutrition source: vitamin D. https://www.hsph.harvard.edu/nutritionsource/vitamin-d/#:~:text=RDA%3A%20The%20Recommended%20Dietary%20Allowance,IU%20(20%20mcg)%20daily.

[44] Wacker M, Holick MF (2013). Sunlight and vitamin D: a global perspective for health. Dermatoendocrinol.

[45] Ross AC, Taylor CL, Yaktine AL (2011). Overview of vitamin D. Dietary Reference Intakes for Vitamin D and Calcium.

[46] Schmid A, Walther B (2013). Natural vitamin D content in animal products. Adv Nutr.

[47] Lehmann U, Gjessing HR, Hirche F (2015). Efficacy of fish intake on vitamin D status: a meta-analysis of randomized controlled trials. Am J Clin Nutr.

[48] Cardwell G, Bornman JF, James AP, Black LJ (2018). A review of mushrooms as a potential source of dietary vitamin D. Nutrients.

[49] Alayed Albarri EM, Sameer Alnuaimi A, Abdelghani D (2022). Effectiveness of vitamin D2 compared with vitamin D3 replacement therapy in a primary healthcare setting: a retrospective cohort study. Qatar Med J.

[50] Janoušek J, Pilařová V, Macáková K (2022). Vitamin D: sources, physiological role, biokinetics, deficiency, therapeutic use, toxicity, and overview of analytical methods for detection of vitamin D and its metabolites. Crit Rev Clin Lab Sci.

[51] Schümmer T, Stangl GI, Wätjen W (2021). Safety assessment of vitamin D and its photo-isomers in UV-irradiated baker’s yeast. Foods.

[52] Bikle DD (2014). Vitamin D metabolism, mechanism of action, and clinical applications. Chem Biol.

[53] Jones G (2018). The discovery and synthesis of the nutritional factor vitamin D. Int J Paleopathol.

[54] Jukic AM, Hoofnagle AN, Lutsey PL (2018). Measurement of vitamin D for epidemiologic and clinical research: shining light on a complex decision. Am J Epidemiol.

[55] Botelho J, Machado V, Proença L, Delgado AS, Mendes JJ (2020). Vitamin D deficiency and oral health: a comprehensive review. Nutrients.

[56] Fraser WD, Tang JC, Dutton JJ, Schoenmakers I (2020). Vitamin D measurement, the debates continue, new analytes have emerged, developments have variable outcomes. Calcif Tissue Int.

[57] Uwitonze AM, Razzaque MS (2018). Role of magnesium in vitamin D activation and function. J Am Osteopath Assoc.

[58] Gil Á, Plaza-Diaz J, Mesa MD (2018). Vitamin D: classic and novel actions. Ann Nutr Metab.

[59] Fleet JC (2017). The role of vitamin D in the endocrinology controlling calcium homeostasis. Mol Cell Endocrinol.

[60] Morris HA, Anderson PH (2010). Autocrine and paracrine actions of vitamin D. Clin Biochem Rev.

[61] Charoenngam N, Holick MF (2020). Immunologic effects of vitamin D on human health and disease. Nutrients.

[62] Carlberg C (2019). Vitamin D signaling in the context of innate immunity: focus on human monocytes. Front Immunol.

[63] Samuel S, Sitrin MD (2008). Vitamin D's role in cell proliferation and differentiation. Nutr Rev.

[64] Umar M, Sastry KS, Chouchane AI (2018). Role of vitamin D beyond the skeletal function: a review of the molecular and clinical studies. Int J Mol Sci.

[65] Ricca C, Aillon A, Bergandi L, Alotto D, Castagnoli C, Silvagno F (2018). Vitamin D receptor is necessary for mitochondrial function and cell health. Int J Mol Sci.

[66] Voltan G, Cannito M, Ferrarese M, Ceccato F, Camozzi V (2023). Vitamin D: an overview of gene regulation, ranging from metabolism to genomic effects. Genes (Basel).

[67] Zmijewski MA, Carlberg C (2020). Vitamin D receptor(s): in the nucleus but also at membranes?. Exp Dermatol.

[68] Hii CS, Ferrante A (2016). The non-genomic actions of Vitamin D. Nutrients.

[69] Żmijewski MA (2022). Nongenomic activities of vitamin D. Nutrients.

[70] Hernigou P, Sitbon J, Dubory A, Auregan JC (2019). Vitamin D history part III: the "modern times"-new questions for orthopaedic practice: deficiency, cell therapy, osteomalacia, fractures, supplementation, infections. Int Orthop.

[71] Wimalawansa SJ (2023). Physiological basis for using vitamin D to improve health. Biomedicines.

[72] Carlberg C (2019). Vitamin D: a micronutrient regulating genes. Curr Pharm Des.

[73] Dimitrov V, Barbier C, Ismailova A (2021). Vitamin D-regulated gene expression profiles: species-specificity and cell-specific effects on metabolism and immunity. Endocrinology.

[74] Ustianowski Ł, Ustianowska K, Gurazda K, Rusiński M, Ostrowski P, Pawlik A (2023). The role of vitamin C and vitamin D in the pathogenesis and therapy of periodontitis-narrative review. Int J Mol Sci.

[75] Diachkova E, Trifonova D, Morozova E (2021). Vitamin D and its role in oral diseases development. Scoping review. Dent J (Basel).

[76] Hovsepian S, Amini M, Aminorroaya A, Amini P, Iraj B (2011). Prevalence of vitamin D deficiency among adult population of Isfahan City, Iran. J Health Popul Nutr.

[77] Palacios C, Gonzalez L (2014). Is vitamin D deficiency a major global public health problem?. J Steroid Biochem Mol Biol.

[78] Md Isa Z, Mohd Nordin NR, Mahmud MH, Hashim S (2022). An update on vitamin D deficiency status in Malaysia. Nutrients.

[79] Holick MF, Binkley NC, Bischoff-Ferrari HA (2011). Evaluation, treatment, and prevention of vitamin D deficiency: an Endocrine Society clinical practice guideline. J Clin Endocrinol Metab.

[80] Sudfeld CR, Mugusi F, Muhihi A (2020). Efficacy of vitamin D(3) supplementation for the prevention of pulmonary tuberculosis and mortality in HIV: a randomised, double-blind, placebo-controlled trial. Lancet HIV.

[81] Thacher TD, Clarke BL (2011). Vitamin D insufficiency. Mayo Clin Proc.

[82] Reichrath J, März W, DE Gruijl FR (2022). An appraisal to address health consequences of vitamin D deficiency with food fortification and supplements: time to act!. Anticancer Res.

[83] Lhamo Y, Chugh PK, Gautam SR, Tripathi CD (2017). Epidemic of vitamin D deficiency and its management: awareness among Indian medical undergraduates. J Environ Public Health.

[84] Cui A, Zhang T, Xiao P, Fan Z, Wang H, Zhuang Y (2023). Global and regional prevalence of vitamin D deficiency in population-based studies from 2000 to 2022: a pooled analysis of 7.9 million participants. Front Nutr.

[85] Taba M Jr, Souza SL, Mariguela VC (2012). Periodontal disease: a genetic perspective. Braz Oral Res.

[86] Kinane DF, Stathopoulou PG, Papapanou PN (2017). Periodontal diseases. Nat Rev Dis Primers.

[87] Laird E, Ward M, McSorley E, Strain JJ, Wallace J (2010). Vitamin D and bone health; potential mechanisms. Nutrients.

[88] Dominguez LJ, Farruggia M, Veronese N, Barbagallo M (2021). Vitamin D sources, metabolism, and deficiency: available compounds and guidelines for its treatment. Metabolites.

[89] Thomas DR (2006). Vitamins in aging, health, and longevity. Clin Interv Aging.

[90] Brancaccio M, Mennitti C, Cesaro A (2022). The biological role of vitamins in athletes’ muscle, heart and microbiota. Int J Environ Res Public Health.

[91] Kumar P, Kumar M, Bedi O (2021). Role of vitamins and minerals as immunity boosters in COVID-19. Inflammopharmacology.

[92] Wang H, Chen W, Li D, Yin X, Zhang X, Olsen N, Zheng SG (2017). Vitamin D and chronic diseases. Aging Dis.

[93] Berezowska M, Coe S, Dawes H (2019). Effectiveness of vitamin D supplementation in the management of multiple sclerosis a systematic review. Int J Mol Sci.

[94] Bilezikian JP, Formenti AM, Adler RA (2021). Vitamin D: dosing, levels, form, and route of administration: does one approach fit all?. Rev Endocr Metab Disord.

[95] Li W, Zheng Q, Xu M, Zeng C, Deng X (2023). Association between circulating 25-hydroxyvitamin D metabolites and periodontitis: Results from the NHANES 2009-2012 and Mendelian randomization study. J Clin Periodontol.

[96] Chapple IL, Mealey BL, Van Dyke TE (2018). Periodontal health and gingival diseases and conditions on an intact and a reduced periodontium: Consensus report of workgroup 1 of the 2017 World Workshop on the Classification of Periodontal and Peri-Implant Diseases and Conditions. J Periodontol.

[97] Chapple IL, Mealey BL, Van Dyke TE (2018). Periodontal health and gingival diseases and conditions on an intact and a reduced periodontium: Consensus report of workgroup 1 of the 2017 World Workshop on the Classification of Periodontal and Peri-Implant Diseases and Conditions. J Clin Periodontol.

[98] Alsulaimani L, Alqarni H, Akel M, Khalifa F (2023). The orthodontics-periodontics challenges in integrated treatment: a comprehensive review. Cureus.

[99] Dixon D, Hildebolt CF, Miley DD (2009). Calcium and vitamin D use among adults in periodontal disease maintenance programmes. Br Dent J.

[100] Harpenau LA, Cheema AT, Zingale JA, Chambers DW, Lundergan WP (2011). Effects of nutritional supplementation on periodontal parameters, carotenoid antioxidant levels, and serum C-reactive protein. J Calif Dent Assoc.

[101] Hiremath VP, Rao CB, Naik V, Prasad KVV (2011). The optimum serum vitamin D level needed to initiate anti-inflammatory effect on gingivitis: a dose-response randomized controlled trial. J Indian Assoc Public Health Dent.

[102] Woelber JP, Bremer K, Vach K (2016). An oral health optimized diet can reduce gingival and periodontal inflammation in humans - a randomized controlled pilot study. BMC Oral Health.

[103] Tennert C, Reinmuth AC, Bremer K (2020). An oral health optimized diet reduces the load of potential cariogenic and periodontal bacterial species in the supragingival oral plaque: a randomized controlled pilot study. Microbiologyopen.

[104] Garcia MN, Hildebolt CF, Miley DD (2011). One-year effects of vitamin D and calcium supplementation on chronic periodontitis. J Periodontol.

[105] Chung HM, Park JY, Ko KA, Kim CS, Choi SH, Lee JS (2022). Periodontal probing on digital images compared to clinical measurements in periodontitis patients. Sci Rep.

[106] Tonetti MS, Sanz M (2019). Implementation of the new classification of periodontal diseases: decision-making algorithms for clinical practice and education. J Clin Periodontol.

[107] Ramenzoni LL, Lehner MP, Kaufmann ME, Wiedemeier D, Attin T, Schmidlin PR (2021). Oral diagnostic methods for the detection of periodontal disease. Diagnostics (Basel).

[108] Schnettler B, Lobos G, Miranda-Zapata E, Denegri M, Ares G, Hueche C (2017). Diet quality and satisfaction with life, family life, and food-related life across families: a cross-sectional pilot study with mother-father-adolescent triads. Int J Environ Res Public Health.

[109] Kandel S (2019). An evidence-based look at the effects of diet on health. Cureus.

[110] Singh A, Singh D (2023). The Paleolithic diet. Cureus.

[111] Karmore UP, Ukey UU, Sharma SK (2023). Effect of dietary modification and physical activity on obese young adults going to gym for weight loss in central India: a before and after study. Cureus.

[112] Wansink B, Wansink A (2022). MyPlate, half-plate, and no plate: how visual plate-related dietary benchmarks influence what food people serve. Cureus.

[113] Nagaria T D, Shinde R K, Shukla S, Acharya S, Acharya N, Jogdand SD (2023). The sunlight-vitamin D Connection: implications for patient outcomes in the surgical intensive care unit. Cureus.

[114] Santra S, Sharma K, Dash I, Mondal S, Mondal H (2022). Bone mineral density, serum calcium, and vitamin D levels in adult thalassemia major patients: experience from a single center in eastern India. Cureus.

[115] Khan AW, Zadran N, Khan A, Ishaq M, Kumar J, Ibrar A, Tahir A (2020). Vitamin D levels and bone mineral density in premenopausal women compared to postmenopausal women: a multi-centre study from Pakistan. Cureus.

[116] Nimavat N, Singh S, Patel D (2022). The relationship between vitamin D levels and severity in illness in COVID-19 patients: a cross-sectional study. Cureus.

[117] Khanolkar S, Hirani S, Mishra A, Vardhan S, Hirani S, Prasad R, Wanjari M (2023). Exploring the role of vitamin D in atherosclerosis and its impact on cardiovascular events: a comprehensive review. Cureus.

[118] Meher A, Goel M, Jain R, Dhadse N, Paiwal K (2023). Vitamin D deficiency and gingival enlargement: a case report. Cureus.

[119] Youssef DA, Miller CW, El-Abbassi AM, Cutchins DC, Cutchins C, Grant WB, Peiris AN (2011). Antimicrobial implications of vitamin D. Dermatoendocrinol.

[120] Bikle DD (2021). Vitamin D: production, metabolism, and mechanisms of action. Endotext [Internet].

[121] Golpour A, Bereswill S, Heimesaat MM (2019). Antimicrobial and immune-modulatory effects of vitamin D provide promising antibiotics-independent approaches to tackle bacterial infections - lessons learnt from a literature survey. Eur J Microbiol Immunol (Bp).

[122] Vargas Buonfiglio LG, Cano M, Pezzulo AA, Vanegas Calderon OG, Zabner J, Gerke AK, Comellas AP (2017). Effect of vitamin D(3) on the antimicrobial activity of human airway surface liquid: preliminary results of a randomised placebo-controlled double-blind trial. BMJ Open Respir Res.

[123] Meghil MM, Cutler CW (2023). Influence of vitamin D on periodontal inflammation: a review. Pathogens.

[124] Jagelavičienė E, Vaitkevičienė I, Šilingaitė D, Šinkūnaitė E, Daugėlaitė G (2018). The relationship between vitamin D and periodontal pathology. Medicina (Kaunas).

[125] Hildebolt CF (2005). Effect of vitamin D and calcium on periodontitis. J Periodontol.

[126] Kim H, Shin MH, Yoon SJ (2020). Low serum 25-hydroxyvitamin D levels, tooth loss, and the prevalence of severe periodontitis in Koreans aged 50 years and older. J Periodontal Implant Sci.

[127] Bikle DD (2012). Vitamin D and bone. Curr Osteoporos Rep.

[128] Álvarez-Mercado AI, Mesa MD, Gil Á (2023). Vitamin D: role in chronic and acute diseases. Encyclopedia of Human Nutrition.

[129] Bui FQ, Almeida-da-Silva CL, Huynh B (2019). Association between periodontal pathogens and systemic disease. Biomed J.

[130] Wei R, Christakos S (2015). Mechanisms underlying the regulation of innate and adaptive immunity by vitamin D. Nutrients.

[131] L Bishop E, Ismailova A, Dimeloe S, Hewison M, White JH (2021). Vitamin D and immune regulation: antibacterial, antiviral, anti-inflammatory. JBMR Plus.

[132] Ismailova A, White JH (2022). Vitamin D, infections and immunity. Rev Endocr Metab Disord.

[133] White JH (2022). Emerging roles of vitamin D-induced antimicrobial peptides in antiviral innate immunity. Nutrients.

[134] Stein SH, Livada R, Tipton DA (2014). Re-evaluating the role of vitamin D in the periodontium. J Periodontal Res.

[135] Gombart AF (2009). The vitamin D-antimicrobial peptide pathway and its role in protection against infection. Future Microbiol.

[136] Park K, Elias PM, Oda Y, Mackenzie D, Mauro T, Holleran WM, Uchida Y (2011). Regulation of cathelicidin antimicrobial peptide expression by an endoplasmic reticulum (ER) stress signaling, vitamin D receptor-independent pathway. J Biol Chem.

[137] Checchi L, Montevecchi M, Checchi V, Zappulla F (2009). The relationship between bleeding on probing and subgingival deposits. An endoscopical evaluation. Open Dent J.

[138] Mariotti A, Hefti AF (2015). Defining periodontal health. BMC Oral Health.

[139] Yin K, Agrawal DK (2014). Vitamin D and inflammatory diseases. J Inflamm Res.

[140] Hoe E, Nathanielsz J, Toh ZQ (2016). Anti-inflammatory effects of vitamin D on human immune cells in the context of bacterial infection. Nutrients.

[141] Anand N, Chandrasekaran SC, Rajput NS (2013). Vitamin D and periodontal health: Current concepts. J Indian Soc Periodontol.

[142] Lu EM (2023). The role of vitamin D in periodontal health and disease. J Periodontal Res.

[143] Madi M, Pavlic V, Mongith Alammar S, Mohammad Alsulaimi L, Shaker Alotaibi R, Mohammed AlOtaibi G, Zakaria O (2021). The association between vitamin D level and periodontal disease in Saudi population, a preliminary study. Saudi Dent J.

[144] Liang F, Zhou Y, Zhang Z, Zhang Z, Shen J (2023). Association of vitamin D in individuals with periodontitis: an updated systematic review and meta-analysis. BMC Oral Health.

[145] Amrein K, Scherkl M, Hoffmann M (2020). Vitamin D deficiency 2.0: an update on the current status worldwide. Eur J Clin Nutr.

[146] Aparna P, Muthathal S, Nongkynrih B, Gupta SK (2018). Vitamin D deficiency in India. J Family Med Prim Care.

[147] Saggese G, Vierucci F, Prodam F (2018). Vitamin D in pediatric age: consensus of the Italian Pediatric Society and the Italian Society of Preventive and Social Pediatrics, jointly with the Italian Federation of Pediatricians. Ital J Pediatr.

[148] Khadilkar A, Kajale N, Oza C (2022). Vitamin D status and determinants in Indian children and adolescents: a multicentre study. Sci Rep.

[149] Cook CE, Thigpen CA (2019). Five good reasons to be disappointed with randomized trials. J Man Manip Ther.

[150] Hariton E, Locascio JJ (2018). Randomised controlled trials - the gold standard for effectiveness research: Study design: randomised controlled trials. BJOG.

[151] Mohseni M, Ameri H, Arab-Zozani M (2022). Potential limitations in systematic review studies assessing the effect of the main intervention for treatment/therapy of COVID-19 patients: an overview. Front Med (Lausanne).

